# Bifunctional Bicarbazole-Benzophenone-Based Twisted Donor–Acceptor–Donor Derivatives for Deep-Blue and Green OLEDs

**DOI:** 10.3390/nano13081408

**Published:** 2023-04-19

**Authors:** Prakalp Gautam, Shahnawaz, Iram Siddiqui, Dovydas Blazevicius, Gintare Krucaite, Daiva Tavgeniene, Jwo-Huei Jou, Saulius Grigalevicius

**Affiliations:** 1 Department of Materials Science and Engineering, National Tsing Hua University, No. 101, Section 2, Guangfu Rd., East District, Hsinchu 30013, Taiwan; 2 Department of Polymer Chemistry and Technology, Kaunas University of Technology, Radvilenu Plentas 19, LT50254 Kaunas, Lithuania

**Keywords:** bi-functional D-A-D derivatives, deep-blue emission, phosphorescent and TADF green OLEDs, high-efficiency

## Abstract

Organic light-emitting diodes (OLEDs) have played a vital role in showing tremendous technological advancements for a better lifestyle, due to their display and lighting technologies in smartphones, tablets, television, and automotive industries. Undoubtedly, OLED is a mainstream technology and, inspired by its advancements, we have designed and synthesized the bicarbazole-benzophenone-based twisted donor–acceptor–donor (D-A-D) derivatives, namely DB13, DB24, DB34, and DB43, as bi-functional materials. These materials possess high decomposition temperatures (>360 °C) and glass transition temperatures (~125 °C), a high photoluminescence quantum yield (>60%), wide bandgap (>3.2 eV), and short decay time. Owing to their properties, the materials were utilized as blue emitters as well as host materials for deep-blue and green OLEDs, respectively. In terms of the blue OLEDs, the emitter DB13-based device outperformed others by showing a maximum EQE of 4.0%, which is close to the theoretical limit of fluorescent materials for a deep-blue emission (CIE_y_ = 0.09). The same material also displayed a maximum power efficacy of 45 lm/W as a host material doped with a phosphorescent emitter Ir(ppy)_3_. Furthermore, the materials were also utilized as hosts with a TADF green emitter (4CzIPN) and the device based on DB34 displayed a maximum EQE of 11%, which may be attributed to the high quantum yield (69%) of the host DB34. Therefore, the bi-functional materials that are easily synthesized, economical, and possess excellent characteristics are expected to be useful in various cost-effective and high-performance OLED applications, especially in displays.

## 1. Introduction

Organic light-emitting diodes (OLEDs) technology has outperformed other technologies in recent decades [1,2,3,4,5,6,7,8,9,10]. OLEDs are the ultimate technology for display and are moving rapidly into lighting. At present, there is an intensive need for high-performance deep-blue emitters for full-color displays and solid-state lightings [11,12,13,14,15,16,17,18]. Deep-blue emissions tend to reduce not only the number of pixels required for blue emission but also the cost of the device. For the same purpose, the realization of high-efficiency and stable deep-blue fluorophores is essential, satisfying the National Television System Committee (NTSC) standard [19,20,21,22,23] of the deep-blue Commission International de I’Eclairage (CIE) coordinates (CIE_y_ ≤ 0.1). To realize the same, several phosphorescent-based emitters were developed [24,25,26]. However, as the emission peaks shift towards the deep-blue region, the nonradiative transition rate of metal d-orbitals tends to increase, making it difficult to achieve high efficiency [24,25,26,27]. In order to solve the problem, small-molecule fluorescent materials are being re-developed due to their high color purity and low cost [28].

To date, several reports have presented deep-blue materials based on anthracenes [29,30,31,32], pyrenes [33,34,35,36,37] and fluorenes [38,39,40,41,42,43]. However, these derivatives, especially anthracene, generally exhibit low singlet-exciton yields, which may be caused by strong electron–hole pairing. As a result, they usually suffer from an aggregation-caused quenching (ACQ) effect [44]. This effect diminishes the device efficiency and causes the color purity to deteriorate [44,45,46]. 

Herein, we introduce a series of donor–acceptor–donor (D-A-D) twisted derivatives based on bicarbazole and benzophenone moieties. While the D-A-D architecture approach reduces the radiative lifetime as low as is feasible [47], the twisted D-A-D architecture tends to exhibit good intermolecular charge transfer and a small ∆*E_ST_*. In addition, bicarbazole, due to its high energy absorption and emission capability, was paired with a strong electron-acceptor benzophenone moiety for effective charge confinement within the emission layer [47,48,49,50,51]. We demonstrated that twisted D-A-D derivatives could provide good thermal and morphological stability and prevent the ACQ by diminishing the electron–hole pairing in the solid state [52]. Hence, a series of four compounds, namely DB13, DB24, DB34, and DB43, was synthesized and investigated. DB13 displayed a maximum EQE of 4.0% for a deep-blue emission. Some of the derivatives also performed as good host materials to realize high-efficiency green phosphorescent as well as TADF-based OLEDs. 

## 2. Experimental Section

### 2.1. Instrumentation

Thermogravimetric analysis (TGA) was performed on a TGAQ50 apparatus (Verder Scientific Haan, Haan, Germany). The TGA and DSC curves were recorded in a nitrogen atmosphere at a heating rate of 10 °C/min. Differential scanning calorimetry (DSC) measurements were carried out using a Bruker Reflex II thermos-system (Bruker, Berlin, Germany). UV–visible spectroscopy was performed using an HP-8453 diode array spectrometer (Agilent Technology Inc., Hachioji, Tokyo, Japan) to measure the absorption spectra of the compounds. In addition, the Tauc plot was derived using the absorbance wavelength. Photoluminescence (PL) spectra were recorded using the Aminco-Bowman Series 2 luminescence spectrometer (Agilent Technology Inc., Hachioji, Tokyo, Japan). Low-temperature PL (LTPL) was recorded using a Hitachi F-7000 fluorescence spectrophotometer (Edinburgh Instruments Ltd., Livingston, UK). LTPL was performed at a low temperature of 77K to obtain the singlet energy of the compounds. Cyclic voltammetry (CV) was carried out using the CH instrument CH1604A potentiostat Annatech Co., Ltd., Taipei, Taiwan). The highest occupied molecular orbital (HOMO) levels were calculated using results of the CV measurement. Time-resolved photoluminescent measurements were performed on an Edinburgh instrument spectrometer FLS980 (Edinburgh Instruments Ltd., Livingston, UK) in order to determine the decay time of the compounds. 

### 2.2. Device Fabrication

A pre-patterned ITO glass substrate was utilized for the fabrication of OLED devices. The substrate was cleaned using acetone and isopropyl alcohol (IPA) for 30 min each at 50 and 60 °C, respectively. The substrates were then transferred to the preheated UV chamber for 10 min of UV treatment. The layer deposition took place in a glove box under an inert atmosphere. The hole injection layer (PEDOT:PSS) was spin-coated at 4000 rpm for 20 s and the substrates were heated at 130 °C for 10 min. An emissive layer was then spin-coated on the cooled substrates at 2500 rpm for 20 s. The substrates were then transferred to the thermal evaporation chamber where electron-injection/-transport layer and aluminium cathode were deposited at a vacuum of 10^−6^ torr. The substrates were kept under a vacuum in a mini chamber of the glove box and individually taken for testing. The testing was carried out in a completely dark room under ambient conditions. The current-density–voltage–luminance (J-V-L) characteristics were recorded using a CS-100A luminescence spectrophotometer, while power efficacy–luminance–current characteristics were recorded using a PR-655 spectrophotometer. The Keithley voltmeter was used to measure the current–voltage (I-V) characteristics. The device area was 0.09 cm^−2^. External quantum efficiency (EQE) of the devices was calculated using the method described in the literature [53].

## 3. Result and Discussion

**Figure 1:**
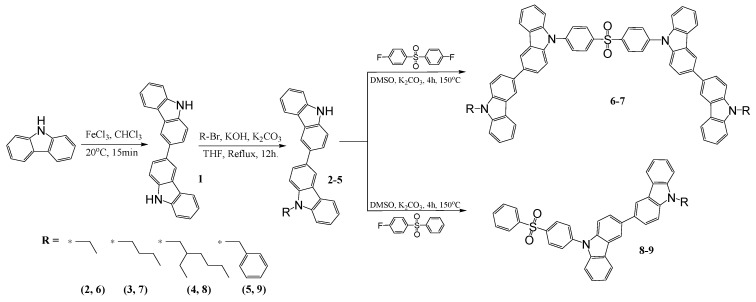
Synthetic pathway of materials DB13 (**7**), DB24 (**8**), DB34 (**9**), and DB43 (**6**).

The synthesis of bicarbazole-based host materials was carried out by the three-step synthetic route as shown in Figure 1. The 3,3′-Bicarbazole (**1**) was obtained by oxidizing carbazole with iron (III) chloride. Various 9-alkyl-9′H-3,3′-bicarbazoles (**2–4**) and 9-benzyl-9′H-3,3′-bicarbazole (**5**) were obtained by the N-alkylation reaction between the bicarbazole **1** and corresponding alkyl or benzyl bromide, using potassium hydroxide and potassium carbonate in tetrahydrofuran (THF). The last step was the nucleophilic aromatic substitution of partially alkylated bicarbazole.

This was calculated using the method described in the literature, with one or two fluorine atoms containing diphenyl sulfone. The mentioned reactions were carried out in DMSO using potassium carbonate as a base and resulted in objective materials DB13 (**7**), DB24 (**8**), DB34 (**9**), and DB43 (**6**). The newly synthesized derivatives were identified by mass spectrometry and NMR spectroscopy. The data were found to be in good agreement with the proposed structure.

**Figure 2:**
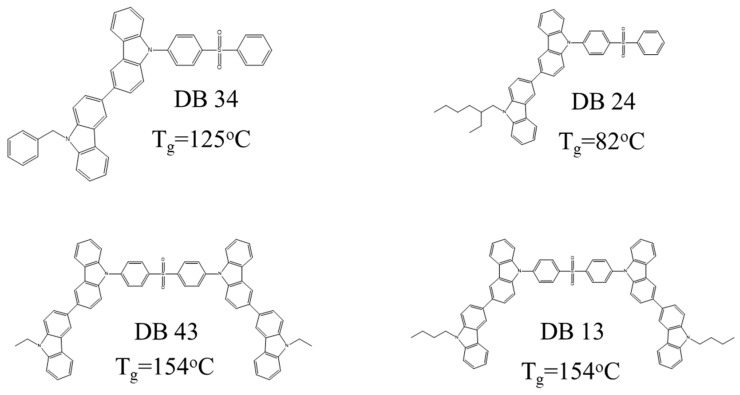
Exact chemical structures of the bicarbazole-benzophenone-based derivatives DB34, DB24, DB43, and DB13.

Figure 2 shows the exact chemical structures of the derivatives in order to demonstrate the different length of alkyl chains in the group DB13 (**7**), DB24 (**8**), and DB43 (**6**). The compound DB34 has in its structure the benzyl fragment. The compounds DB43 and DB13 consist of two 3,3′-bicarbazole units, where DB13 has a longer alkyl chain than that of DB43. Inoue et al. reported the effect of an alkyl chain’s length on the solubility of the organic compounds. The longer the alkyl chain, the better the solubility of the molecule in a suitable solvent [54]. 

### 3.1. Characteristics of the Presented Materials 

#### 3.1.1. DFT Calculations

**Figure 3:**
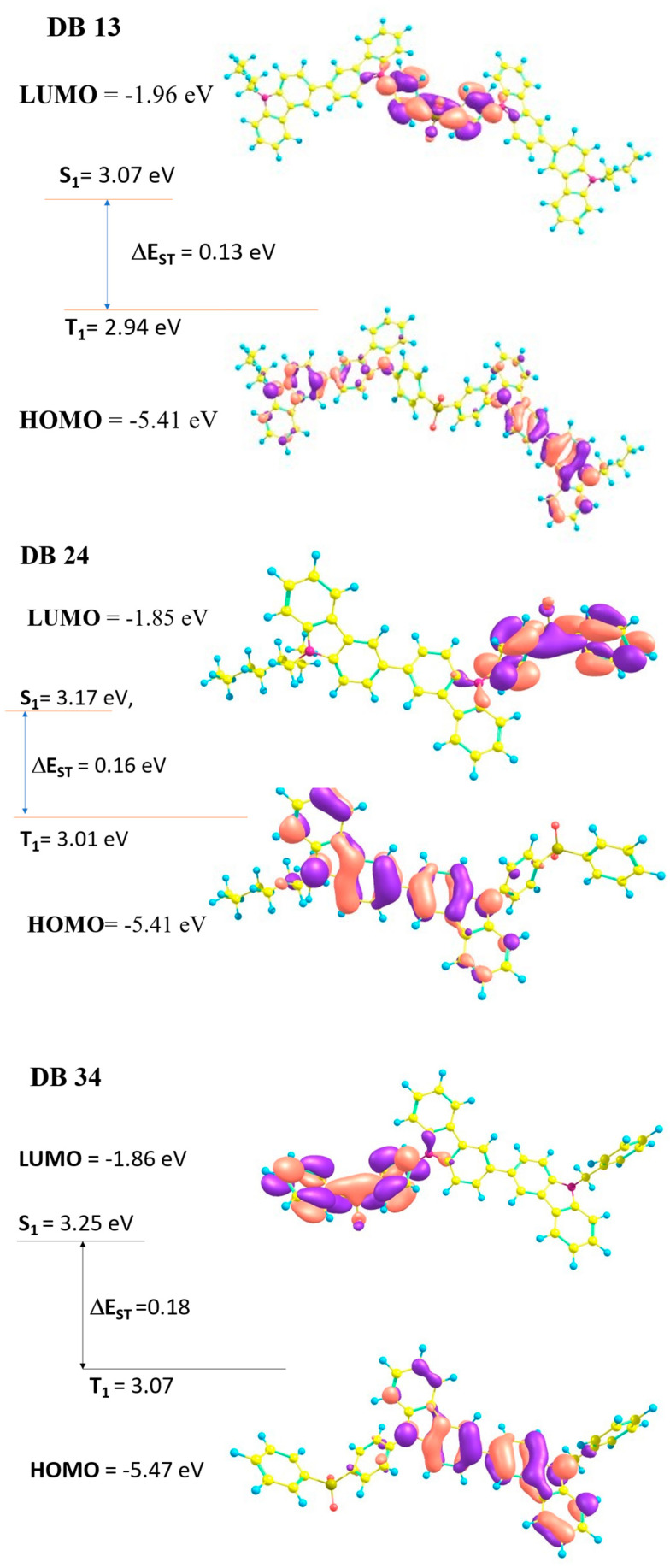

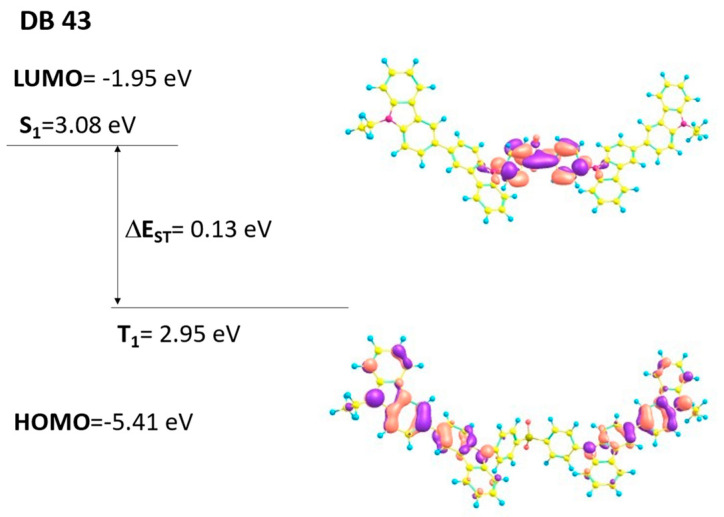
Electron density contours of frontier molecular orbitals (FMO) and HOMO, LUMO, singlet, triplet, and singlet–triplet energy gap electron distribution of the compounds DB13, DB24, DB34, and DB43.

The electron density contours of frontier molecular orbitals (FMO) and HOMO, LUMO, singlet, triplet, and singlet–triplet energy gap electron distribution of the compounds DB13, DB24, DB34, and DB43 are shown in Figure 3 and provided in Table 1. The theoretically measured values of the compounds DB13, DB24, DB34, and DB43 show HOMO, LUMO, and the energy gap between singlet and triplet (Δ*E_ST_*) and singlet and triplet energies. All the materials show a small Δ*E_ST_* (<0.2 eV), referring to the effective utilization of triplet-level excitons. The HOMO and LUMO levels are suitable for developing blue OLEDs and also for use as host materials for OLED devices.

#### 3.1.2. Photophysical Properties

The compounds DB13, DB24, DB34, and DB43 possess a high photoluminescence quantum yield (PLQY) of 50.5, 61.8, 68.5 and 66.5%, respectively. The values of PLQYs are also tabulated in Table 1.

**Figure 4:**
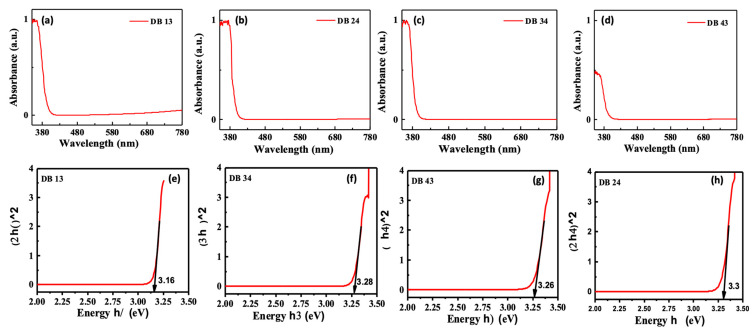
(**a**–**d**) Ultraviolet–visible absorbance (UV abs) spectra and (**e**–**h**) Tauc plot, represent the absorption wavelength and bandgap, respectively, of the compounds DB13, DB24, DB34 and DB43.

The UV abs (Figure 4a–d) of the compounds DB13, DB24, DB34, and DB43 were examined using tetrahydrofuran (THF) solvent under ambient conditions. The spectra of the prepared solutions were recorded using the quartz cuvette. The absorption peaks were observed at around 375 nm for all the compounds. This is understandable, because all the derivatives have the same chromophores in their structures. A Tauc plot (Figure 4e–h) was prepared with the absorption wavelength and intensity, using the following equations:

**x-axis:** *(α × hν)*^1/2^ (1)

**y-axis:** *hν* (2)

where *α* is the absorption coefficient and *hν* is the energy (*hν* = 1240/wavelength).

The maximal absorbance wavelength was utilized as the excitation wavelength for measuring photoluminescence. The excitation wavelengths and bandgap are presented in Table 1. The singlet energies calculated are shown in the Supplementary File (Figure S1).

**Figure 5:**
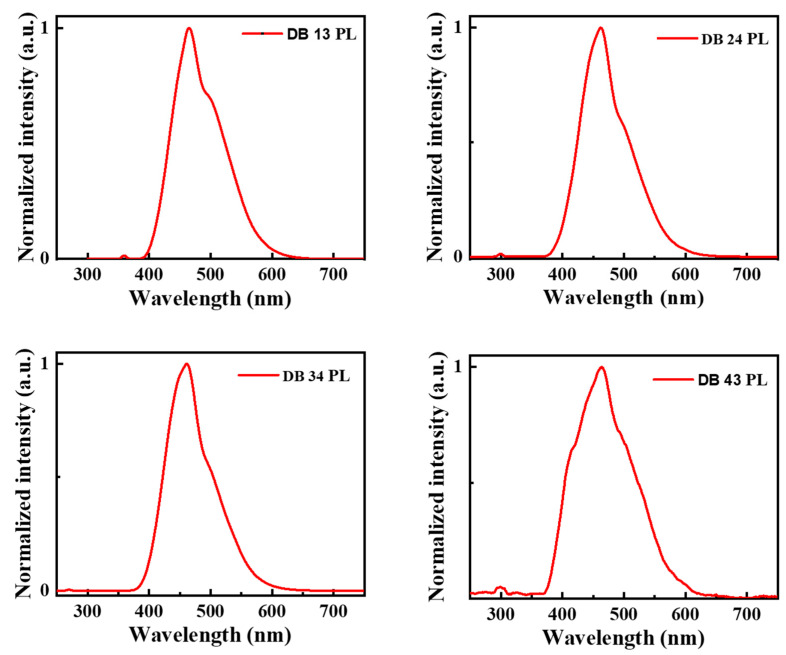
Photoluminescence (PL) spectra of the compounds DB13, DB24, DB34, and DB43.

**Figure 6:**
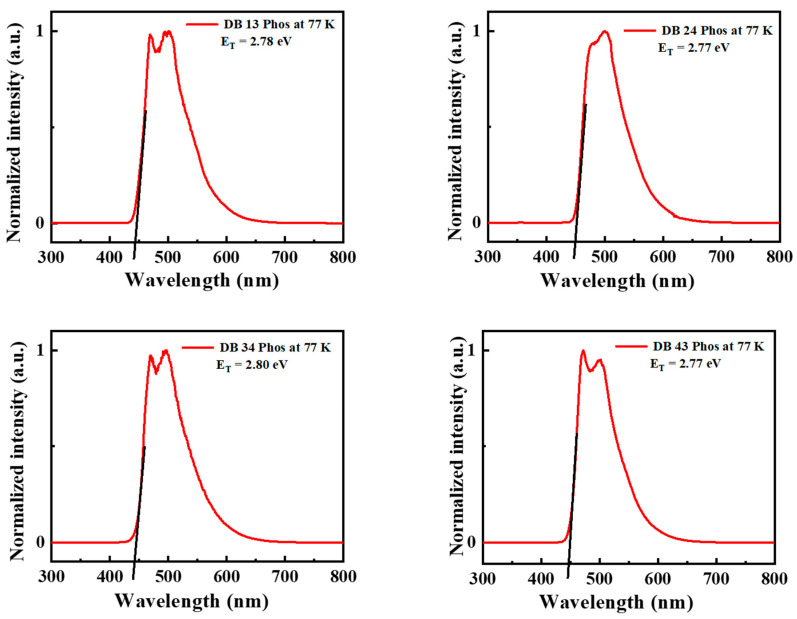
Low–temperature photoluminescence (LTPL) spectra at 77 K of the compounds DB13, DB24, DB34 and DB43.

Figure 5 shows the PL spectra of the compounds DB13, DB24, DB34, and DB43, demonstrating the emission wavelength maxima in the region of 450–470 nm. The low-temperature photoluminescence (LTPL) spectra, which are presented in Figure 6, were also measured to determine the triplet energies. The compounds DB13, DB24, DB34, and DB43 possess high triplet energy levels of 2.78, 2.77, 2.80, and 2.77 eV, respectively, and could be tested as suitable host materials for green phosphorescent emitters, as well as for green TADF emitters. The values of triplet energies are also tabulated in Table 1.

**Figure 7:**
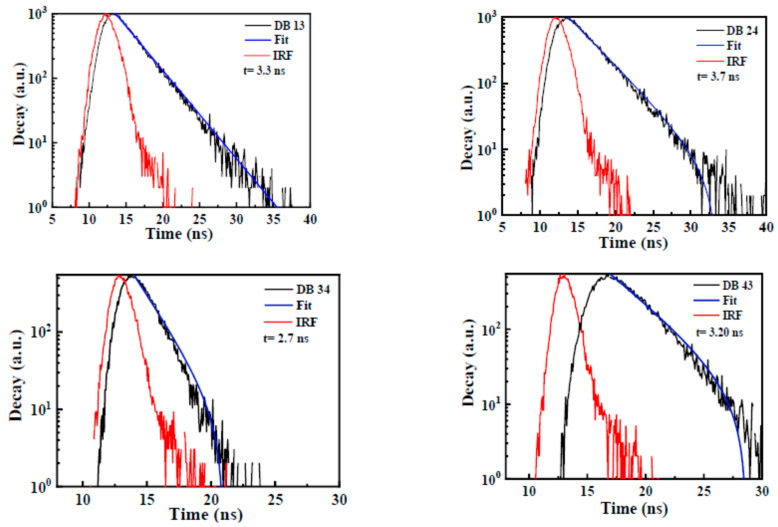
Time-resolved photoluminescence (TRPL) spectra for the PL decay kinetics analysis of the compounds DB 13, DB 24, DB 34, and DB 43.

Figure 7 shows the time-resolved photoluminescence (TRPL) analysis representing the decay time of the compounds. The time values of 3.3, 3.7, 2.7, and 3.2 ns were determined for DB13, DB24, DB34, and DB43, respectively. It could be seen that all the compounds emit radiatively, with a short decay time. The decay values are provided in Table 1. IRF corresponds to the instrument response function that was measured before and after each measurement as a control parameter.

#### 3.1.3. Electrochemical Properties

**Figure 8:**
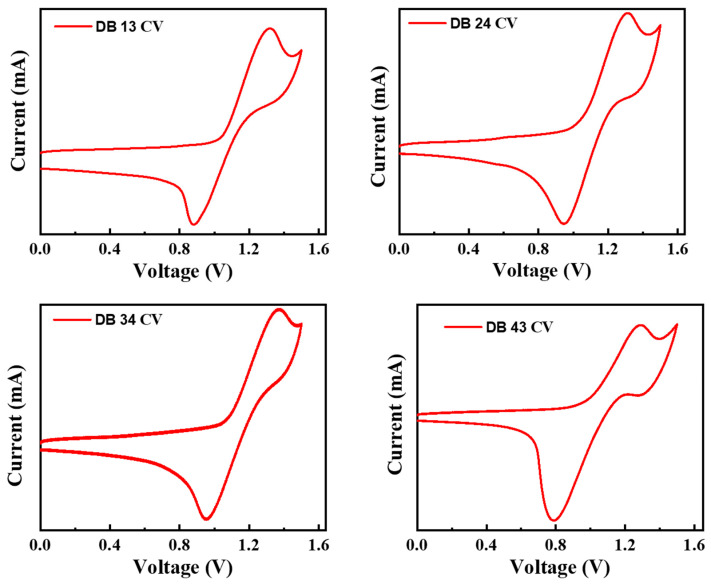
Cyclic voltammetry scans for calculation of HOMO levels of the compounds DB13, DB24, DB34 and DB43.

Electrochemical characteristics of the compounds DB 13, DB 24, DB 34, and DB 43 were estimated using cyclic voltammetry (CV) measurements, which are shown in Figure 8.

The HOMO levels were calculated using Equation (3):

(3) $$E_{\mathrm{HOMO}}=-[4.4+E_{onset}^{ox}]$$

The LUMO levels were calculated using the following Equation (4):

E_LUMO_ = E_HOMO_ + *E*_g_ (4)

where *E_g_* is the bandgap calculated using the Tauc plot. The calculated HOMO energy levels were found to be −5.73, −5.71, −5.77, and −5.69 eV and the LUMO levels were found to be −2.57, −2.41, −2.49, and −2.43 eV for, correspondingly, DB 13, DB 24, DB 34, and DB 43. The bandgap was obtained using the plot of Equations (1) and (2) and was found to be 3.16, 3.30, 3.28, and 3.26 eV for the compounds DB 13, DB 24, DB 34 and DB 43, respectively (Table 1). The HOMO and LUMO levels of the derivatives were found to be very suitable for blue emitters, as well as for host materials.

#### 3.1.4. Thermal Properties

**Figure 9:**
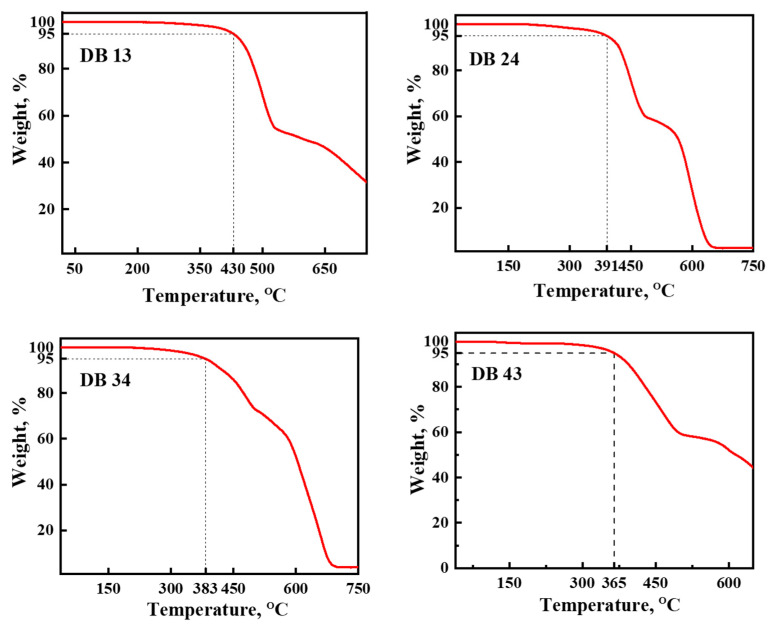
Data of thermogravimetric analysis (TGA) of the compounds DB13, DB24, DB34 and DB43.

The behavior under heating of the synthesized materials DB13, DB24, DB34, and DB43 was studied using DSC and TGA under a nitrogen atmosphere. It was established that the objective compounds demonstrate very high thermal stability. Data from the TGA analyses are shown in Figure 9. The temperatures of a 5% weight loss (*T_d_*) for derivatives DB13, DB24, DB34, and DB43 were 365 °C, 430 °C, 391 °C and 383 °C, respectively, as confirmed by TGA at a heating rate of 10 °C/min (Figure 9). It could be observed that materials DB13 and DB43 have slightly lower thermal stability, probably due to the presence of two alkyl chains in their structures.

**Figure 10:**
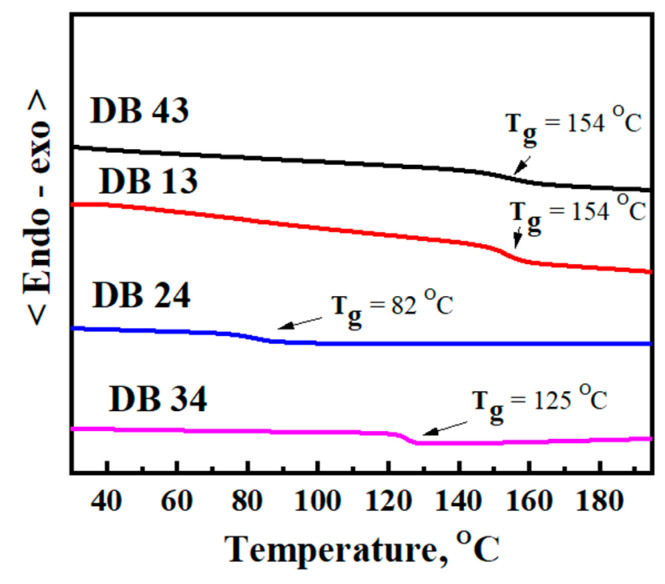
DSC curves of second heating for estimating glass transition temperatures (*T_g_*) of the compounds DB13, DB24, DB34, and DB43.

The DSC curves of the second heating of compounds DB13, DB24, DB34, and DB43 are presented in Figure 10. It can be clearly seen from the curves that some of the new derivatives have very high glass transition temperatures (*T_g_*), of 154 °C for DB13, and also 154 °C for DB34 and 125 °C for DB43. The material DB24 demonstrated a lower glass transition temperature of 82 °C, due to the branched 2-ethylhexyl group, which is in the structure of the derivative and decreases its morphological stability. Therefore, the TGA and DSC results confirm that many of the materials are well suited for application in the amorphous electroactive layers of OLED devices.

Table 1 shows the photophysical, electrochemical and thermal properties of the compounds DB13, DB24, DB34, and DB43. The table demonstrates that the compounds possess high photoluminescence yields, high decomposition temperatures and also high glass transition temperatures for derivatives DB13, DB34 and DB43. The compounds also have large bandgaps, which are suitable for the application of the materials in OLEDs as an emitter as well as a host. 

### 3.2. Structure and Characterization of Electroluminescent OLED Devices

**Figure 11:**
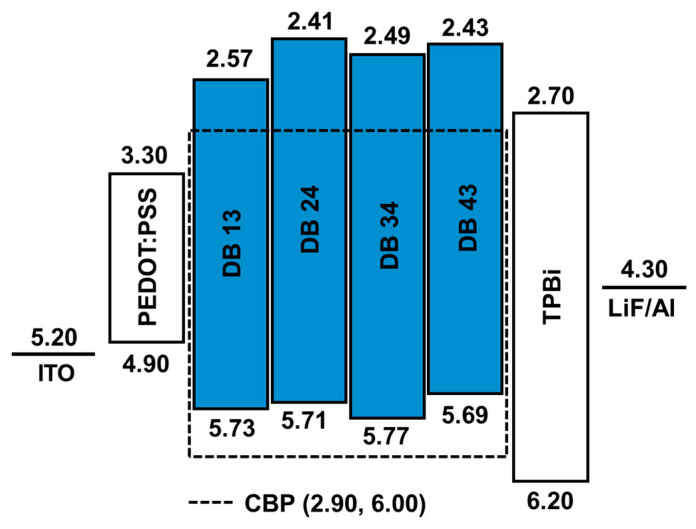
Energy-level diagram in eV of the solution-processed blue OLED devices containing emitters DB 13, DB 24, DB 34, and DB 43 doped in CBP host material.

The schematic energy-level diagram in eV of blue OLED devices fabricated in this work by utilizing the emitters DB13, DB24, DB34 and DB43 doped in the commercial 4,4′-Bis(N-carbazolyl)-1,1′-biphenyl (CBP) host matrix is shown in Figure 11. The fabricated devices were composed as doped devices, having the structure: ITO (125 nm)/PEDOT:PSS (35 nm)/host: (x wt%) emitter (x = 5.0, 10, and 15%) (20 nm)/TPBi (40 nm)/LiF (1 nm)/Al (200 nm). 

**Figure 12:**
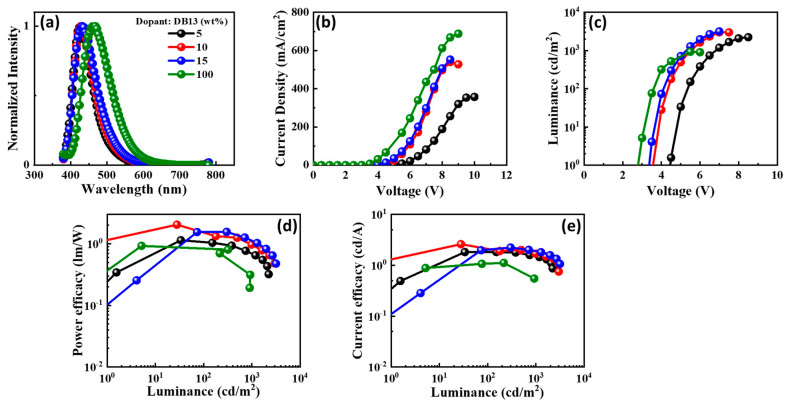
The electroluminescent (EL) properties of the device with emitter DB13 doped in CBP host matrix at varying concentrations showing (**a**) EL spectra, (**b**) current density–voltage, (**c**) luminance–voltage, (**d**) power efficacy–luminance, and (**e**) current efficacy–luminance characteristics.

**Figure 13:**
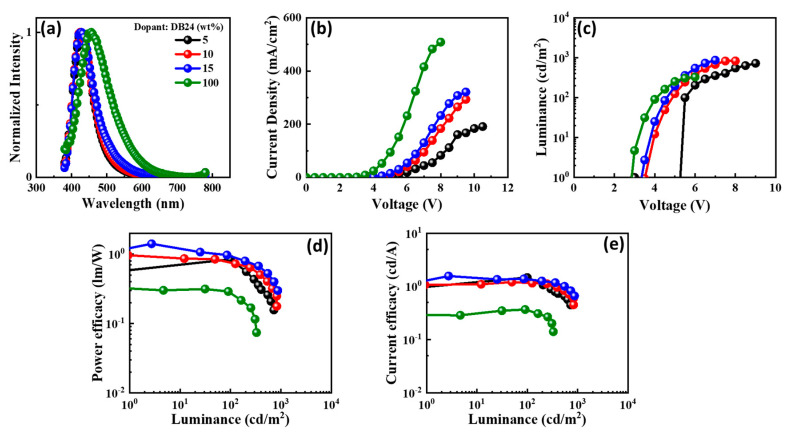
The electroluminescent (EL) properties of the device with emitter DB24 doped in CBP host matrix at varying concentrations showing (**a**) EL spectra, (**b**) current density–voltage, (**c**) luminance–voltage, (**d**) power efficacy–luminance, and (**e**) current efficacy–luminance characteristics.

**Figure 14:**
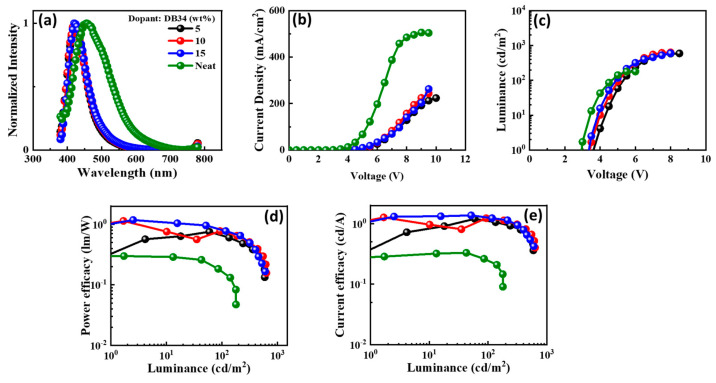
The electroluminescent (EL) properties of the device with emitter DB34 doped in CBP host matrix at varying concentrations showing (**a**) EL spectra, (**b**) current density–voltage, (**c**) luminance–voltage, (**d**) power efficacy–luminance, and (**e**) current efficacy–luminance characteristics.

**Figure 15:**
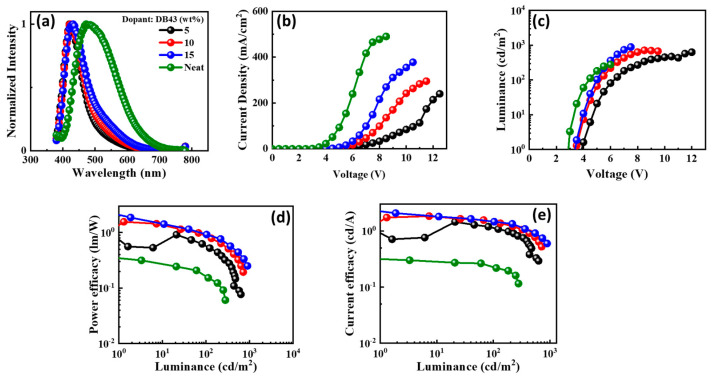
The electroluminescent (EL) properties of the device with emitter DB43 doped in CBP host matrix at varying concentrations showing (**a**) EL spectra, (**b**) current density–voltage, (**c**) luminance–voltage, (**d**) power efficacy–luminance, and (**e**) current efficacy–luminance characteristics.

The electroluminescence (EL) spectra of the emitters doped in the CBP host matrix, and the characteristics of the devices are shown in Figure 12, Figure 13, Figure 14 and Figure 15 and also provided in Table 2. Each figure shows (a) the EL spectra, (b) current-density–voltage, (c) luminance–voltage, (d) power-efficacy–luminance and (e) current-efficacy–luminance characteristics. 

It can be seen from Figure 12, Figure 13, Figure 14 and Figure 15 that the EL spectra of emitters DB13, DB24, DB34, and DB43 peak in the region 430–450 nm, with an indication of the blue emission. The presence of a single peak indicates the complete host–guest energy transfer. The EL emission wavelength of the devices was close to the PL spectra of the used emitter, indicating the origin of emission from the material. Both doped and non-doped devices showed a similar EL emission peak. Figure 12, Figure 13, Figure 14 and Figure 15 also show the characteristics of current-density–luminance-voltage and power-efficacy–luminance–current-efficacy. The non-doped devices showed a current density higher than that of the doped devices and the efficacies were lower than those of the doped devices. Therefore, the role of the host was significant and a 10 wt% DB13 emitter-based device outperformed other devices by displaying a maximum power efficacy (PE_max_) of 2.0 lm/W, and current efficacy (CE_max_) of 2.5 cd/A with a turn-on voltage of 3.7 eV. Moreover, the DB13-based device also displayed the highest EQE_max_ of 4.0%, which is close to the theoretical limit of fluorescent emitters for a deep-blue emission, with a CIE_y_ of 0.09. This device demonstrated a higher EQE as compared with many other fluorescent emitters which are reviewed in the scientific literature [55]. The DB13-based fluorescent OLED displayed an even deeper blue emission and better performance than the recently reported device, based on the hybridized local and charge-transfer (HLCT) mechanism [56]. As reported, the enhancement was attributed to the LE-dominated HLCT state; however, the power and current efficiencies were significantly lower than in this report. The above statement shows that twisted D-A derivatives prove to be better candidates for fluorescent emission.

As shown in Table 2, the device with the DB13 emitter shows the highest PE, CE and EQE among all the devices. The results may be connected to the presence of two alkyl chains in the molecule, which improves the solubility of the material for the wet-processed device fabrication, a high glass-transition temperature (T_g_), high decomposition temperature (T_d_), suitable HOMO and LUMO levels, enabling efficient host–guest energy transfer and the presence of two bicarbazole donor moieties for balanced charge transfer. Moreover, the incorporation of the benzophenone moiety into the chemical structure may have resulted in the deep-blue emission from the OLED device. 

**Figure 16:**
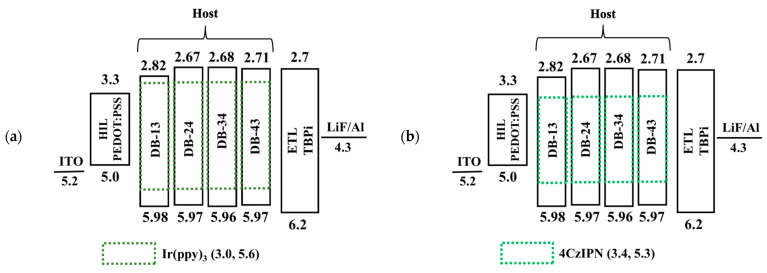
Energy-level diagram in eV of the solution-processed green OLED devices containing hosts DB13, DB24, DB34, and DB43, doped with commercial (**a**) phosphorescent green emitter Ir(ppy)_3_ and (**b**) commercial TADF green emitter 4CzIPN.

Furthermore, owing to the wide bandgap and high triplet energies of the compounds DB13, DB24, DB34 and DB43, they were utilized as host materials for the application in green OLEDs. For the same purpose, the green emitters of generation two and three, i.e., the phosphorescent emitter Ir(ppy)_3_ and TADF emitter 4CzIPN, respectively, were utilized. Figure 16 shows the energy-level diagram in eV of the solution-processed green OLED devices consisting of hosts DB13, DB24, DB34, and DB43, doped with the green commercial phosphorescent emitter Ir(ppy)_3_ and TADF emitter 4CzIPN. The device structure was therefore composed as ITO (125 nm)/PEDOT:PSS (35 nm)/host: (x wt%) emitter (host = DB13, DB24, DB34, and DB43) (emitter = Ir(ppy)_3_ or 4CzIPN) (x = 10, 12.5 and 5% for Ir(ppy)_3_), (x = 1, 3 and 5% for emitter 4CzIPN) (20 nm)/TPBi (40 nm)/LiF (1 nm)/Al (200 nm). 

Figures S2–S4 show the electroluminescent characteristics of devices based on the hosts DB13, DB24 and DB34, doped with the green phosphorescent emitter Ir(ppy)_3_. Figures S2a, S3a and S4a show the EL spectra of the devices peaking at ~540 nm with the green emission. The peak from the EL spectra of the devices is similar to the PL peak of Ir(ppy)_3_, indicating the origin of the emission [57]. The single EL peak resembles the complete host-to-guest energy transfer. Figures S2b–e, S3b–e, and S4b–e show the luminance–voltage and efficiencies curves. Amongst all of these, a 12.5 wt% Ir(ppy)_3_ doped in a DB13-based device showed the best performance, with a PE_max_ of 45 lm/W, CE_max_ of 43 cd/A, EQE_max_ of 10.6%, and L_max_ of 37680 cd/m^2^, with a low roll-off at higher luminance value. 

Table 3 shows the OLED characteristics of green devices based on hosts DB13, DB24, DB34, and DB43. doped with the commercial phosphorescent green emitter Ir(ppy)_3_. The good performance of the device using DB13 may be due to its suitable chemical structure, longer aliphatic chains, high decomposition temperature, small singlet–triplet energy gap (Δ*E_ST_*), i.e., effective triplet exciton utilization, and suitable HOMO-LUMO levels, compared to those of Ir(ppy)_3_, which enabled an efficient host-to-guest energy transfer in the emitting layer. Devices using other compounds have also shown comparable efficiencies. However, the DB43-based OLED has shown poorer performance, which might be attributed to the large Δ*E_ST_,* disabling the use of triplet excitons. 

Figures S5–S7 show the OLED characteristics of devices based on the hosts DB13, DB24 and DB34, doped with the green TADF emitter 4CzIPN. Figures S5a, S6a, and S7a show the EL spectra of the devices peaking at ~530 nm with the green emission. The peak from the EL spectra of the devices is like the PL peak of 4CzIPN, and the bathochromic shift is only observed with increasing doping concentration, indicative of the origin of emission [58]. In addition, the single EL peak resembles the complete host-to-guest energy transfer. Figures S2b–e, S3b–e, and S4b–e show the luminance–voltage and efficiencies curves. Amongst all of these, a device based on a 3wt% 4CzIPN emitter in DB34 host outperformed other devices, with an EQE_max_ of 10.8%, which is higher than that of the many described efficient phosphorescent green devices [59]. However, the PE and luminance are slightly lower than those of the mentioned devices. The possible reason might be attributed to triplet–triplet annihilation (TTA). Further reducing Δ*E_ST_* in the synthesized compounds may enable the effective utilization of triplet excitons and may result in better performance of the TADF-based green OLEDs. Table 4 shows the comprehensive list of OLED characteristics of the described devices using hosts DB13, DB24 and DB34. 

## 4. Conclusions

The newly synthesized bicarbazole-benzophenone-based materials possess a large bandgap and high triplet energy, which support their bifunctionality both as an emitter and a host material for OLED applications. Moreover, the materials demonstrate good thermal and morphological stability with very high decomposition temperatures and also high glass-transition temperatures for some compounds as well as short decay time, as confirmed by high PLQY. Two aliphatic chains having the compound DB13 displayed better device performance than their counterparts, probably due to its better solubility and film-forming properties. The bicarbazole-benzophenone-bicarbazole D-A-D-based emitter displayed an EQE_max_ of 4.0% for a deep-blue emission (CIE_y_ 0.09), which confirms its large potential as a deep-blue emitter. On the other hand the developed emitter has better first-generation OLED characteristics than those of the well-known carbazole-benzophenone-carbazole based device, probably due to its better film-forming properties.

Some of the compounds have also shown promising results as host materials for green phosphorescent as well as TADF-based OLEDs. The host DB13-based device showed a PE_max_ of 45 lm/W and low roll-off for the phosphorescent green OLED, while the host DB34-based TADF device outperformed all the devices, with an EQE_max_ of 10.8%, which is even slightly higher than that of the mentioned phosphorescence-based green devices. These results reveal that some of these cost-effective materials possess excellent photophysical, electrochemical and thermal properties, and can be applicable to a variety of display and solid-state lighting applications, especially in TVs, laptops, desktops, mobile phones, and interior lightings, etc. Moreover, the efficiency of the devices could be enhanced by further reducing Δ*E_ST_* and effectively utilizing triplet-state excitons in order for use in high-power applications such as headlights and street lights, etc. We believe that this research will provide a good pathway for the development of future products for academics as well as for industry.

## Figures and Tables

**Table 1:** The excitation wavelength (*λ_ex_*), emission wavelength (*λ_em_*), bandgap (*E_g_*), PLQY (*Φ*), decay time, theoretical and calculated HOMO-LUMO levels, values of singlet energy (*S*_1_) and triplet energy (*T*_1_), singlet–triplet energy gap (Δ*E_ST_*), decomposition temperatures (*T_d_*) and glass-transition temperatures (*T_g_*) of the compounds DB13, DB24, DB34 and DB43.

Emitter	*λ_ex_* (nm)	*λ_em_* (nm)	*E_g_* (eV)	*Φ* (%)	Decay (ns)	HOMO (eV)		LUMO (eV)		*S*_1_ (eV)		*T*_1_ (eV)		Δ*E_ST_* (eV)		*T_d_* (°C)	*T_g_* (°C)
						Theo.	Cal.	Theo.	Cal.	Theo.	Cal.	Theo.	Cal.	Theo.	Cal.		
**DB 13**	365.2	465.4	3.16	50.5	3.30	5.41	5.73	1.96	2.57	3.07	3.10	2.94	2.78	0.13	0.32	430	154
**DB 24**	375.4	462.5	3.30	61.8	3.70	5.41	5.71	1.85	2.41	3.17	3.17	3.01	2.77	0.16	0.35	391	82
**DB 34**	363.0	462.0	3.28	68.5	2.70	5.47	5.77	1.86	2.49	3.25	3.14	3.07	2.80	0.18	0.34	383	125
**DB 43**	366.9	463.2	3.26	66.5	3.30	5.41	5.69	1.95	2.43	3.08	3.23	2.95	2.77	0.13	0.46	365	154

**Table 2:** Electroluminescent (EL) characteristics of the devices with emitters DB34, DB24, DB43 and DB13 doped in CBP host matrix at varying concentrations, displaying turn-on voltage at luminance >1 cd m^−2^, power efficacy, current efficacy, external quantum efficiency, CIE, and maximal luminance.

Emitter	Concentration (wt%)	Turn-on Voltage (V_on_)	Power Efficacy (lm/W)	Current Efficacy (cd/A)	EQE (%)	CIE	Max Luminance (cd/m^2^)
			@100/1000 cd/m ^2^ and Max				
DB34	5.0	4.2	0.7/-/0.8	1.1/-/1.2	2.3/-/2.3	(0.16, 0.07)/-	589
	10	4.0	0.8/-/0.8	1.2/-/1.2	2.0/-/2.0	(0.16, 0.08)/-	631
	15	3.8	0.8/-/1.0	1.3/-/1.4	1.8/-/2.0	(0.16, 0.09)/-	599
	100	3.4	0.2/-/0.3	0.2/-/0.3	0.1/-/0.2	(0.19, 0.24)/-	179
DB 24	5.0	5.1	0.8/-/0.8	1.5/-/1.5	2.5/-/2.5	(0.16, 0.08)/-	724
	10	3.9	0.8/-/0.8	1.2/-/1.2	1.7/-/1.8	(0.16, 0.09)/-	830
	15	3.7	0.9/-/1.0	1.4/-/1.4	1.6/-/1.6	(0.16, 0.11)/-	867
	100	3.1	0.3/-/0.3	0.4/-/0.4	0.2/-/0.2	(0.19, 0.21)/-	238
DB 43	5.0	4.6	0.6/-/0.9	1.1/-/1.4	1.3/-/1.6	(0.17, 0.11)/-	631
	10	4.1	0.9/-/1.2	1.5/-/1.7	1.6/-/1.4	(0.18, 0.14)/-	715
	15	4.0	0.9/-/1.2	1.5/-/1.6	1.2/-/1.2	(0.19, 0.16)/-	892
	100	3.2	0.2/-/0.3	0.2/-/0.3	0.1/-/0.1	(0.25, 0.34)/-	278
DB 13	5.0	4.6	1.1/0.7/1.1	1.8/1.5/1.8	3.2/2.5/3.4	(0.16, 0.08)/(0.16, 0.08)/-	2230
	10	3.7	1.7/ 1.0/ 2.0	2.3/ 1.7/ 2.5	3.4/2.5/4.0	(0.16, 0.09)/(0.16, 0.09)/-	2987
	15	3.5	1.6/1.1/1.6	2.0/1.9/2.2	2.8/2.5/2.9	(0.16, 0.10)/(0.16, 0.10)/-	3167
	100	3.0	0.9/-/1.0	1.1/-/1.1	0.6/-/0.6	(0.16, 0.2)/-	928

**Table 3:** Electroluminescent (EL) characteristics of the devices with hosts DB13, DB24, DB34, and DB43, doped with green phosphorescent emitter Ir(ppy)_3_ at varying concentrations, displaying turn-on voltage, power efficacy, current efficacy, external quantum efficiency, CIE, and luminance.

Host	Ir(ppy)_3_ Doping con. (wt%)	Turn-on Voltage (V)	PE_max_/CE_max_/EQE_max_ (lm/W/cd/A/%)	PE_100_/CE_100_/EQE_100_ (lm/W/cd/A/%)	PE_1,000_/CE_1000_/EQE_1000_ (lm/W/cd/A/%)	PE_10,000_/CE_10,000_/EQE_10,000_ (lm/W/cd/A/%)	CIE_xy_ Coordinates	Maxi. Lum. (cd/m^2^)
**DB 13**	10	2.6	40.0/41.1/11.1	39.8/40.6/11.0	32.1/40.9/11.1	17.2/ 32.8/ 8.9	(0.32, 0.62)/(0.32, 0.62)/(0.32, 0.62)	33,870
	12.5	3.1	45.4/43.4/ 10.6	40.0/43.3/ 10.6	33.4/ 42.5 /10.5	20.8/ 36.4/ 8.7	(0.31, 0.62)/ (0.31, 0.62)/ (0.31, 0.63)	37,680
	15	2.5	34.6/35.3/ 9.5	34.6/33.7/ 9.1	28.7/33.5/ 9.4	17.5/ 29.5/ 7.9	(0.32, 0.62)/ (0.32, 0.62)/ (0.32, 0.62)	32,300
**DB 24**	10	2.8	37.8/32.5/ 9.0	30.9/32.4/ 8.9	24.2/30.9/ 8.5	11.4/ 21.8/ 6.0	(0.32, 0.62)/ (0.31, 0.62) (0.31, 0.62)	1410
	12.5	2.9	30.8/28.3/ 7.8	28.7/28.4/ 7.8	21.2/27.0/ 7.4	9.5 /18.2/ 5.0	(0.32, 0.62)/ (0.31, 0.62)/ (0.31, 0.62)	16,960
	15	2.8	25.7/26.2/ 7.2	23.3/26.0/ 7.2	16.8/24.1/ 6.6	6.4/ 14.3/ 3.9	(0.32, 0.62)/ (0.32, 0.62)/ (0.31, 0.62)	16,200
**DB 34**	10	2.6	37.9/36.2/ 9.8	33/ 34.7/ 9.4	27.1/34.5/ 9.3	12.8/ 24.5/ 6.6	(0.32, 0.62)/ (0.32, 0.62)/ (0.32, 0.62)	22,570
	12.5	2.8	32.7/32.8/ 8.7	31.6/32.2/ 8.7	24.3/30.9/ 8.3	9.8/ 18.7/ 5.1	(0.31, 0.62)/ (0.31, 0.62)/ (0.31, 0.63)	21,430
	15	2.9	20.2/21.0/ 5.7	18.7/20.8/ 5.6	14.5/20.8/ 5.6	7.3/ 15.1/ 4.1	(0.32, 0.62)/ (0.32, 0.62)/ (0.32, 0.62)	22,100

**Table 4:** Electroluminescent (EL) characteristics of the devices with hosts DB13, DB24 and DB34, doped with green commercial TADF emitter 4CzIPN at varying concentrations, displaying turn-on voltage, power efficacy, current efficacy, external quantum efficiency, CIE, and maximal luminance.

Host	4CzIPN Doping con. (wt%)	Turn-on Voltage (V)	PE_max_/ CE_max_/EQE_max_ (lm/W/cd/A/ %)	PE_100_/CE_100_/EQE_100_ (lm/W/cd/A/ %)	PE_1,000_/CE_1000_/EQE_1000_ (lm/W/cd/A/ %)	CIE_xy_ Coordinates	Maxi. Lum. (cd/m^2^)
**DB 13**	1	3.2	16.6/15.8/4.8	7.9/11.1/3.6	4.1/8.5/3.1	(0.32, 0.51)/(0.29, 0.46)	3108
	3	3.8	24.9/22.2/6.7	12.1/15.5/4.7	2.3/4.8/1.5	(0.32, 0.55)/(0.29, 0.51)	5869
	5	2.9	16.2/15.7/4.7	14.1/15.7/4.7	6.6/11.7/3.6	(0.35, 0.55)/(0.34, 0.54)	712
**DB 24**	1	3.1	-/-/-	10.1/12.9/4.6	3.7/7.0/2.7	(0.29, 0.47)/(0.27, 0.42)	2823
	3	2.8	22.9/23.1/7.7	20.7/23.1/7.7	12.1/17.3/5.8	(0.31, 0.52)/(0.30, 0.51)	6231
	5	2.8	18.4/20.5/6.6	18.4/20.5/6.6	10.8/15.5/5.0	(0.31, 0.54)/(0.31, 0.53)	7689
**DB 34**	1	2.7	30.4/27.1/8.9	21.3/23.1/7.7	10.2/14.7/5.0	(0.27, 0.51)/(0.26, 0.49)	3782
	3	2.6	37.5/33.5/10.8	32.0/32.6/10.6	15.8/22.6/7.4	(0.30, 0.54)/(0.29, 0.53)	9484
	5	2.6	34.7/33.2/10.5	34.7/33.2/10.5	18.9/24.0/7.6	(0.30, 0.55)/(0.29, 0.55)	12480

## Data Availability

The data presented in this study are available on request from the corresponding authors.

## Supplementary Materials

The following supporting information can be downloaded at: https://www.mdpi.com/article/10.3390/nano13081408/s1, Figure S1: singlet energy calculation using the intercept of PL and absorbance wavelength, Figure S2: the electroluminescent (EL) properties of the emitter Ir(ppy)_3_ doped in DB 13 host matrix at varying concentrations, Figure S3: the electroluminescent (EL) properties of the emitter Ir(ppy)_3_ doped in DB 24 host matrix at varying concentrations, Figure S4: the electroluminescent (EL) properties of the emitter Ir(ppy)_3_ doped in DB 34 host matrix at varying concentrations, Figure S5: the electroluminescent (EL) properties of the emitter 4CzIPN doped in DB 23 host matrix at varying concentrations, Figure S6: the electroluminescent (EL) properties of the emitter 4CzIPN doped in DB 23 host matrix at varying concentrations, Figure S7: The electroluminescent (EL) properties of the emitter 4CzIPN doped in DB 34 host matrix at varying concentrations, description of experiments of synthesis with references [60,61].
