# Supplementary material for: Bifunctional Bicarbazole-Benzophenone-Based Twisted Donor–Acceptor–Donor Derivatives for Deep-Blue and Green OLEDs
Source: Nanomaterials (Basel). 2023 Apr 19;13(8):1408. doi: 10.3390/nano13081408 (PMC10146648; doi:10.3390/nano13081408)
Supplement: Supplementary file 1 [file nanomaterials-13-01408-s001.zip › nanomaterials-2172095-supplementary.pdf]

Supplementary Materials

Bifunctional Bicarbazole-Benzophenone-Based Twisted Donor–Acceptor–Donor Derivatives for Deep-Blue and Green OLEDs

Prakalp Gautam 1,†, Shahnawaz 1,†, Iram Siddiqui 1,†, Dovydas Blazevicius 2,†, Gintare Krucaite 2, Daiva Tavgeniene 2 , Jwo-Huei Jou 1,* and Saulius Grigalevicius 2,*

[1] Department of Materials Science and Engineering, National Tsing Hua University, No. 101, Section 2, Guangfu Rd., East District, Hsinchu 30013, Taiwan

[2] Department of Polymer Chemistry and Technology, Kaunas University of Technology, Radvilenu Plentas 19, LT50254 Kaunas, Lithuania

* Correspondence: jjou@mx.nthu.edu.tw (J.-H.J.); saulius.grigalevicius@ktu.lt (S.G.)

† These authors contributed equally to this work.

Table of Content

1. Synthesis
2. Singlet Energy
3. Electroluminescence
4. References

Synthesis

Carbazole, benzylbromide, bromoethane, 2-ethylhexylbromide, 1-bromobutane, $FeCl_3$, KOH, K_2CO_3, Na_2SO_4, bis(4-fluorophenyl) sulfone, 4-fluorophenyl phenyl sulfone, butan-2-one, chloroform, DMSO and THF were purchased from Aldrich and used as received.

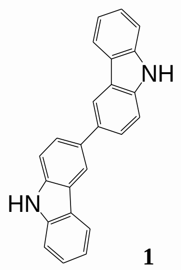

1

3,3′-Bicarbazole (**1**) was obtained by oxidation of carbazole using $FeCl_3$ as it was described earlier [1].

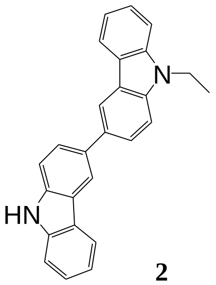

2

9-Ethyl-9′H-3,3′-bicarbazole (**2**). Bromoethane (0.66 g, 6.02 mmol) was added to a stirred solution of 3,3′-bicarbazole (**1**) (2.00 g, 6.02 mmol) in 50 mL of tetrahydrofuran. The mixture was heated to reflux, and then potassium carbonate (1.66 g, 12.04 mmol) and powdered potassium hydroxide (2.02 g, 36.12 mmol) was added stepwise. The resulting mixture was left to react for 4 h. After TLC control, the inorganic salts were filtered off and the product was purified by silica gel column chromatography using tetrahydrofuran/hexane (vol. ratio 1:3) as an eluent. Yield: 0.59 g (27%) of yellowish material.

^{1}H NMR (400 MHz, $CDCl_3$-*d*$_6$, δ, m.d.): 8.45 (dd, 2H, J1 = 10.8 Hz, J2 = 1.2 Hz), 8.25 (d, 1H, J = 7.6 Hz), 8.22 (d, 1H, J = 7.6 Hz), 8.05 (s, 1H), 7.87 (dd, 1H, J1 = 8.4 Hz, J2 = 2.0 Hz), 7.83 (dd, 1H, J1 = 8.4 Hz, J2 = 1.6 Hz), 7.56-7.45 (m, 6H), 7.31 (t, 2H, 7.6 Hz), 4.44 (q, 2H, J = 7.2 Hz), 1.51 (t, 3H, J = 7.2 Hz). 13C NMR (400 MHz, CDCl3-d6, , m.d.): 140.45, 140.03, 139.11, 138.56, 134.14, 133.38, 125.94, 125.86, 125.75, 125.60, 124.00, 123.61, 123.55, 123.20, 120.57, 120.46, 119.50, 119.09, 118.92, 118.85, 110.81, 110.74, 108.69, 108.60, 37.68, 13.90.

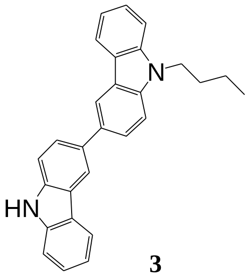

3

9-Butyl-9′H-3,3′-bicarbazole (**3**). 1-Bromobutane (0.82 g, 6.02 mmol) was added to a stirred solution of 3,3′-bicarbazole (**1**) (2.00 g, 6.02 mmol) in 50 mL of tetrahydrofuran. The mixture was heated to reflux, and then potassium carbonate (1.66 g, 12.04 mmol) and powdered potassium hydroxide (2.02 g, 36.12 mmol) was added stepwise. The resulting mixture was left to react for 4 h. After TLC control, the inorganic salts were filtered off and the product was purified by silica gel column chromatography using tetrahydrofuran/hexane (vol. ratio 1:5) as an eluent. Yield: 0.96 g (41%) of yellowish material.

^{1}H NMR (400 MHz, $CDCl_3$-*d_6*, δ, m.d.): 8.29 (s, 2H), 8.11–8.06 (m, 2H), 7.73–7.67 (m, 2H), 7.41–7.32 (m, 6H), 7.17 (t, 2H, J = 7.2 Hz), 4.29 (t, 2H, J = 7.2 Hz), 1.79 (quint, 2H, J = 7.2 Hz), 1.38–1.29 (m, 2H), 0.87 (t, 3H, J = 7.2 Hz). ^{13}C NMR (400 MHz, $CDCl_3$-*d_6*, δ, m.d.): 139.64, 138.56, 133.33, 125.94, 125.86, 125.72, 125.57, 124.00, 123.61, 123.43, 123.08, 120.51, 120.46, 119,51, 119.03, 119.00, 118.92, 118.82, 110.83, 110.77, 108.95, 108.86, 42.99, 31.25, 20.65, 13.97. MS (APCI$^+$, 20 V): 339.10 ([M+H], 100%).

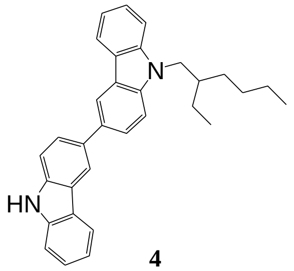

4

9-(2-Ethylhexyl)-9′H-3,3′-bicarbazole (**4**) was synthesized by a similar procedure that was described earlier [2]. 2-Ethylhexylbromide (1.16 g, 6.02 mmol) was added to a stirred solution of 3,3′-bicarbazole (**1**) (2.00 g, 6.02 mmol) in 50 mL of tetrahydrofuran. The mixture was heated to reflux, and then potassium carbonate (1.66 g, 12.04 mmol) and powdered potassium hydroxide (2.02 g, 36.12 mmol) was added stepwise. The resulting mixture was left to react for 4 h. After TLC control, the inorganic salts were filtered off and the product was purified by silica gel column chromatography using tetrahydrofuran/hexane (vol. ratio 1:7) as an eluent. Yield: 1.28 g (48%) of yellowish material.

^{1}H NMR (400 MHz, $CDCl_3$-*d_6*, δ, m.d.): 8.47 (d, 2H, J = 7.2 Hz), 8.26 (d, 1H, J = 7.6 Hz), 8.23 (d, 1H, J = 7.6 Hz), 7.88 (d, 1H, J = 7.2 Hz), 7.84 (dd, 1H, J_1 = 8.4 Hz, J_2 = 1.6 Hz), 7.57–7.43 (m, 6H), 7.33 (t, 2H, J = 7.2 Hz), 4.25 (t, 2H, J = 6.4 Hz), 2.21–2.14 (m, 1H), 1.51–1.32 (m, 8H), 1.00 (t, 3H, J = 7.4 Hz), 0.95 (t, 3H, J = 7.2 Hz). ^{13}C NMR (400 MHz, $CDCl_3$-*d_6*, δ, m.d.): 141.45, 140.14, 138.55, 133.28, 125.95, 125.86, 125.71, 125.56, 124.00, 123.61, 123.40, 123.07, 120.46, 119.52, 118.91, 118.80, 110.84, 110.77, 109.25, 109.15, 47.57, 39.52, 31.09, 28.91, 24.48, 23.14, 14.13, 10.99. MS (APCI$^+$, 20 V): 444.67 ([M+H], 100%).

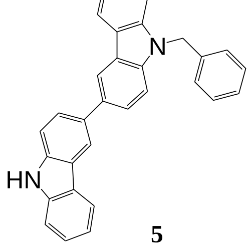

5

9-Benzyl-9′H-3,3′-bicarbazole (**5**). Benzylbromide (1.02 g, 6.02 mmol) was added to a stirred solution of 3,3′-bicarbazole (**1**) (2.00 g, 6.02 mmol) in 50 mL of tetrahydrofuran. The mixture was heated to reflux,

and then potassium carbonate (1.66 g, 12.04 mmol) and powdered potassium hydroxide (2.02 g, 36.12 mmol) was added stepwise. The resulting mixture was left to react for 4 h. After TLC control, the inorganic salts were filtered off and the product was purified by silica gel column chromatography using tetrahydrofuran/hexane (vol. ratio 1:3) as an eluent. Yield: 0.74 g (29%) of yellowish material.

^{1}H NMR (400 MHz, $CDCl_3$-*d*$_6$, δ, m.d.): 8.36 (s, 1H), 8.31 (s, 1H), 8.15 (d, 1H, J = 7.6 Hz), 8.09 (d, 1H, J = 7.6 Hz), 8.01 (s, 1H), 7.71 (d, 2H, J = 8.4 Hz), 8.45 (d, 1H, J = 8.4 Hz), 7.38 (t, 4H, J = 6.4 Hz), 7.34–7.31 (m, 1H), 7.22–7.17 (m, 5H), 7.12 (d, 2H, J = 7.2 Hz), 5.50 (s, 2H). ^{13}C NMR (400 MHz, $CDCl_3$-*d*$_6$, δ, m.d.): 141.15, 140.01, 139.82, 138.57, 137.22, 133.81, 128.83, 127.50, 126.47, 125.96, 125.95, 125.84, 125.81, 124.00, 123.63, 123.59, 123.27, 120.52, 120.44, 119.52, 119.28, 119.05, 118.95, 110.79, 110.72, 109.14, 109.04, 46.72. MS (APCI^{+}, 20 V): 443.66 ([M+H], 100%).

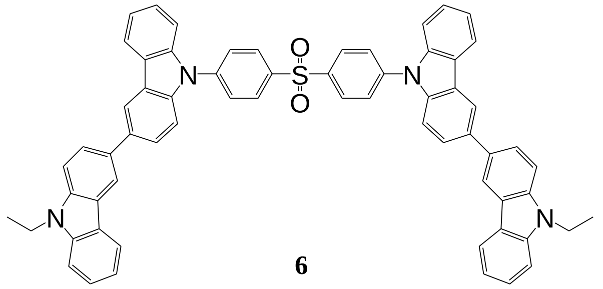

6

Bis(4-(9-Ethyl-3,3′-bicarbazol-9′-yl)phenyl)sulphone (**6**). 9-Ethyl-9′H-3,3′-bicarbazole (**2**) (0.40 g, 1.11 mmol), potassium carbonate (1.53 g, 11.10 mmol) and bis(4-fluorophenyl) sulfone (0.14 g, 0.56 mmol) were stirred in 10 mL of DMSO at 150 °C under nitrogen for 4 h. After TLC control the reaction mixture was cooled and quenched by the addition of ice water. The product was extracted by chloroform. The combined extract was dried over anhydrous Na_2SO_4. The crude product was purified by silica gel column chromatography using the mixture of THF and hexane (vol. ratio 1:3) as an eluent. Yield: 0.46 g (89%) of white amorphous material. T_g = 154 °C (DSC).

^{1}H NMR (400 MHz, $CDCl_3$-*d*$_6$, δ, m.d.): 8.47 (dd, 4H, J_1 = 10.0 Hz, J_2 = 1.6 Hz), 8.33 (d, 2H, J = 8.4 Hz), 8.28–8.23 (m, 4H), 7.90 (d, 2H, J = 8.4 Hz), 7.86 (dd, 2H, J_1 = 8.8 Hz, J_2 = 1.6 Hz), 7.83 (dd, 2H, J_1 = 8.6 Hz, J_2 = 1.8 Hz), 7.66–7.58 (m, 4H), 7.56–7.47 (m, 12H), 7.40 (t, 2H, J = 7.4 Hz), 7.30 (t, 2H, J = 7.0 Hz), 4.45 (q, 4H, J = 7.6 Hz), 1.51 (t, 6H, J = 7.0 Hz). ^{13}C NMR (400 MHz, $CDCl_3$-*d*$_6$, δ, m.d.): 142.89, 140.47, 140.40, 139.32, 139.26, 138.96, 135.74, 132.72, 129.79, 127.10, 126.49, 126.21, 125.86, 125.48, 124.72, 124.33, 123.60, 123.12, 121.12, 120.72, 120.57, 119.07, 118.94, 118.88, 109.92, 109.78, 108.78, 108.65, 37.69, 13.89. MS (APCI^{+}, 20 V): 935.34 ([M+H], 100%).

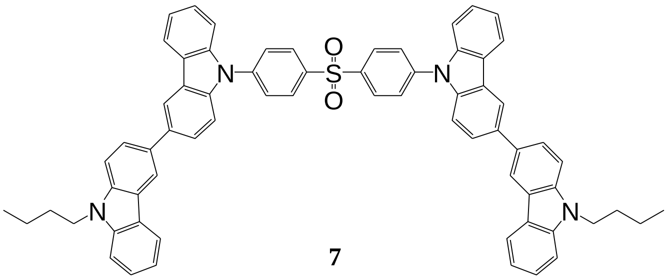

7

Bis(4-(9-Butyl-3,3′-bicarbazol-9′-yl)phenyl)sulphone (**7**). 9-Butyl-9′H-3,3′-bicarbazole (**3**) (0.40 g, 1.03 mmol), potassium carbonate (1.42 g, 10.30 mmol) and bis(4-fluorophenyl) sulfone (0.13 g, 0.51 mmol) were stirred in 10 mL of DMSO at 150 °C under nitrogen for 4 h. After TLC control the reaction mixture was cooled and quenched by the addition of ice water. The product was extracted by chloroform. The combined extract was dried over anhydrous Na_2SO_4. The crude product was purified by silica gel column chromatography using the mixture of THF and hexane (vol. ratio 1:3) as an eluent. Yield: 0.42 g (82%) of white amorphous material. T_g = 154 °C (DSC).

^{1}H NMR (400 MHz, $CDCl_3$-*d*$_6$, δ, m.d.): 8.46 (dd, 4H, J_1 = 13.2 Hz, J_2 = 1.6 Hz), 8.4 (d, 4H, J = 8.8 Hz), 8.28–8.22 (m, 4H), 7.91 (d, 4H, J = 8.8 Hz), 7.84 (dt, 4H, J_1 = 8.8 Hz, J_2 = 1.6 Hz), 7.63 (d, 2H, J = 8.4 Hz), 7.59–7.46 (m, 10H), 7.40 (t, 2H, J = 7.4 Hz), 7.31–7.27 (m, 2H), 4.39 (t, 4H, J = 7.2 Hz), 1.94 (quint, 4H, J = 7.4 Hz), 1.52–1.42 (m, 4H), 1.00 (t, 6H, J = 7.4 Hz). ^{13}C NMR (400 MHz, $CDCl_3$-*d*$_6$, δ, m.d.): 142.88, 140.96, 140.39, 139.77, 139.33, 138.95, 135.74, 132.65, 129.80, 127.10, 126.48, 126.21, 125.81, 125.43, 124.70, 124.32, 123.46, 122.98, 121.11, 120.71, 120.49, 119.07, 118.99, 118.88, 109.92, 109.77, 109.01, 108.88, 43.00, 31.21, 20.62, 13.94. MS (APCI$^+$, 20 V): 991.16 ([M+H], 100%).

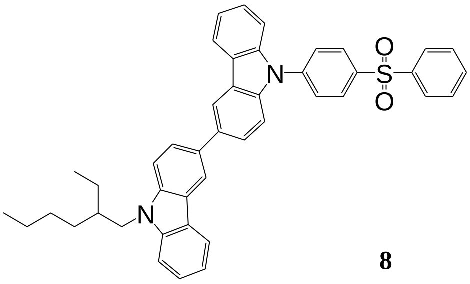

8

4-(9-(2-Ethylhexyl)-3,3′-bicarbazol-9′-yl)phenyl) phenyl sulphone (**8**). 9-(2-Ethylhexyl)-9′H-3,3′-bicarbazole (**4)** (0.40 g, 0.91 mmol), potassium carbonate (1.26 g, 9.10 mmol) and 4-fluorophenyl phenyl sulfone (0.22 g, 0.91 mmol) were stirred in 10 mL of DMSO at 150 °C under nitrogen for 4 h. After TLC control the reaction mixture was cooled and quenched by the addition of ice water. The product was extracted by chloroform. The combined extract was dried over anhydrous Na_2SO_4. The crude product

was purified by silica gel column chromatography using the mixture of THF and hexane (vol. ratio 1:3) as an eluent. Yield: 0.58 g (96%) of white amorphous material. T_g = 82 °C (DSC).

^{1}H NMR (400 MHz, $CDCl_3$-*d6*, δ, m.d.): 8.45 (d, 2H, J = 12.4 Hz), 8.26–8.22 (m, 4H), 8.10 (d, 2H, J = 8.0 Hz), 7.84–7.79 (m, 4H), 7.68–7.60 (m, 3H), 7.57–7.45 (m, 6H), 7.38 (t, 1H, J = 7.0 Hz), 7.31–7.28 (m, 1H), 4.23 (d, 2H, J = 4.8 Hz), 1.47–1.30 (m, 9H), 0.99–0.91 (m, 6H). ^{13}C NMR (400 MHz, $CDCl_3$-*d6*, δ, m.d.): 142.59, 141.43, 140.41, 140.24, 139.65, 138.97, 135.63, 133.49, 132.63, 129.62, 129.52, 127.88, 126.97, 126.42, 126.15, 125.77, 125.40, 124.63, 124.26, 123.41, 122.95, 121.02, 120.66, 120.41, 119.01, 118.89, 118.84, 109.88, 109.75, 109.28, 109.16, 47.57, 39.49, 31.07, 28.88, 24.45, 23.10, 14.09, 10.96. MS ($APCI^+$, 20 V): 660.46 ([M+H], 100%).

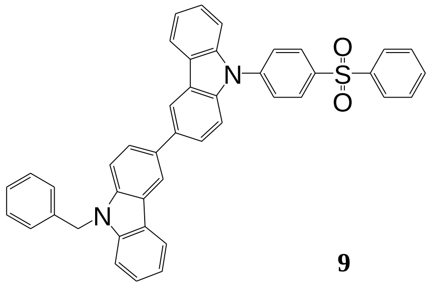

9

4-(9-Benzyl-3,3′-bicarbazol-9′-yl)phenyl) phenyl sulphone (**9**). 9-Benzyl-9′H-3,3′-bicarbazole (**5**) (0.40 g, 0.95 mmol), potassium carbonate (1.31 g, 9.50 mmol) and 4-fluorophenyl phenyl sulfone (0.23 g, 0.95 mmol) were stirred in 10 mL of DMSO at 150 °C under nitrogen for 4 h. After TLC control the reaction mixture was cooled and quenched by the addition of ice water. The product was extracted by chloroform. The combined extract was dried over anhydrous Na_2SO_4. The crude product was purified by silica gel column chromatography using the mixture of THF and hexane (vol. ratio 1:3) as an eluent. Yield: 0.55 g (91%) of white amorphous material. T_g = 125 °C (DSC).

^{1}H NMR (400 MHz, $CDCl_3$-*d6*, δ, m.d.): 8.45 (dd, 2H, J_1 = 8.6 Hz, J_2 = 1.4 Hz), 8.26–8.21 (m, 4H), 8.12–8.09 (m, 2H), 7.82–7.77 (m, 4H), 7.67–7.62 (m, 3H), 7.56–7.36 (m, 8H), 7.34–7.27 (m, 3H), 7.22–7.20 (m, 2H), 5.58 (s, 2H). ^{13}C NMR (400 MHz, $CDCl_3$-*d6*, δ, m.d.): 142.56, 141.42, 141.18, 140.40, 139.96, 139.66, 139.00, 137.16, 135.51, 134.49, 133.50, 133.20, 129.77, 129.62, 129.52, 129.03, 128.84, 127.88, 127.54, 126.97, 126.46, 126.15, 126.09, 125.71, 124.62, 124.22, 123.68, 123.19, 121.03, 120.66, 120.54, 120.43, 119.38, 119.04, 109.90, 109.76, 109.25, 109.11, 46.71. MS ($APCI^+$, 20 V): 638.38 ([M+H], 100%).

Singlet Energy

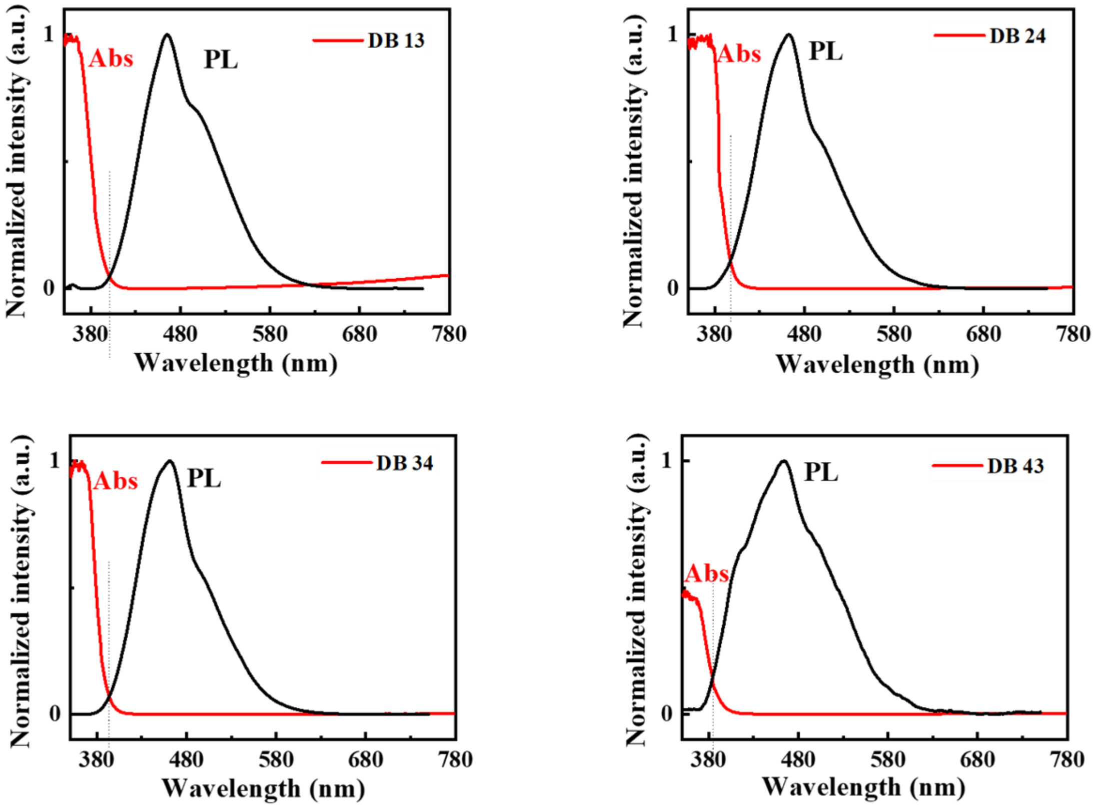

Figure S1. Singlet energy calculation using the intercept of PL and absorbance wavelength.

Electroluminescence

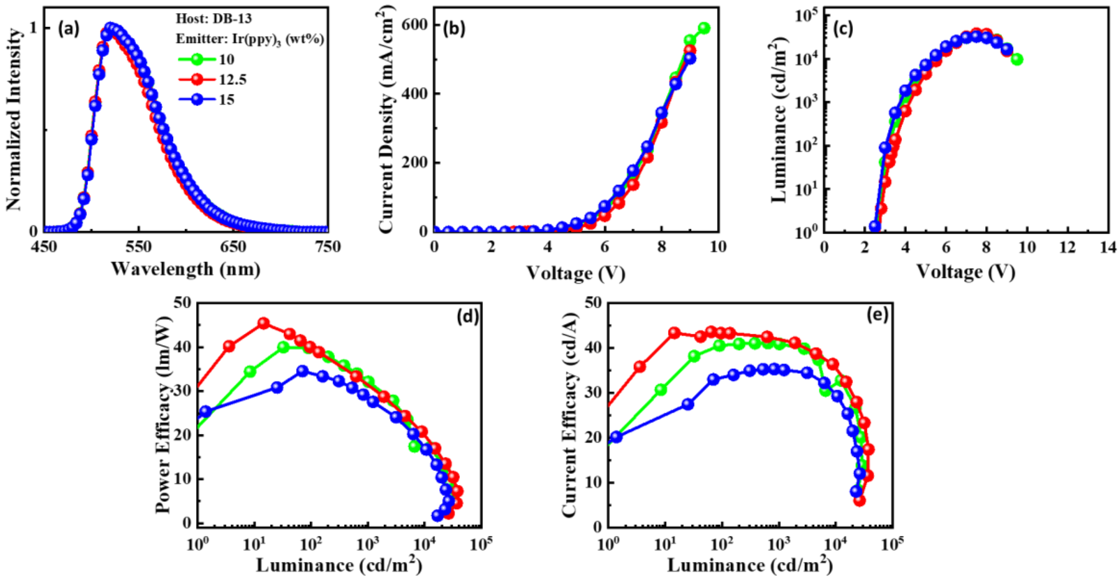

Figure S2. The electroluminescent (EL) properties of the emitter $Ir(ppy)_3$ doped in DB 13 host matrix at varying concentrations showing (**a**) EL spectra, (**b**) current density-voltage, (**c**) luminance-voltage, (**d**) power efficacy-luminance, and (**e**) current efficacy-luminance characteristics.

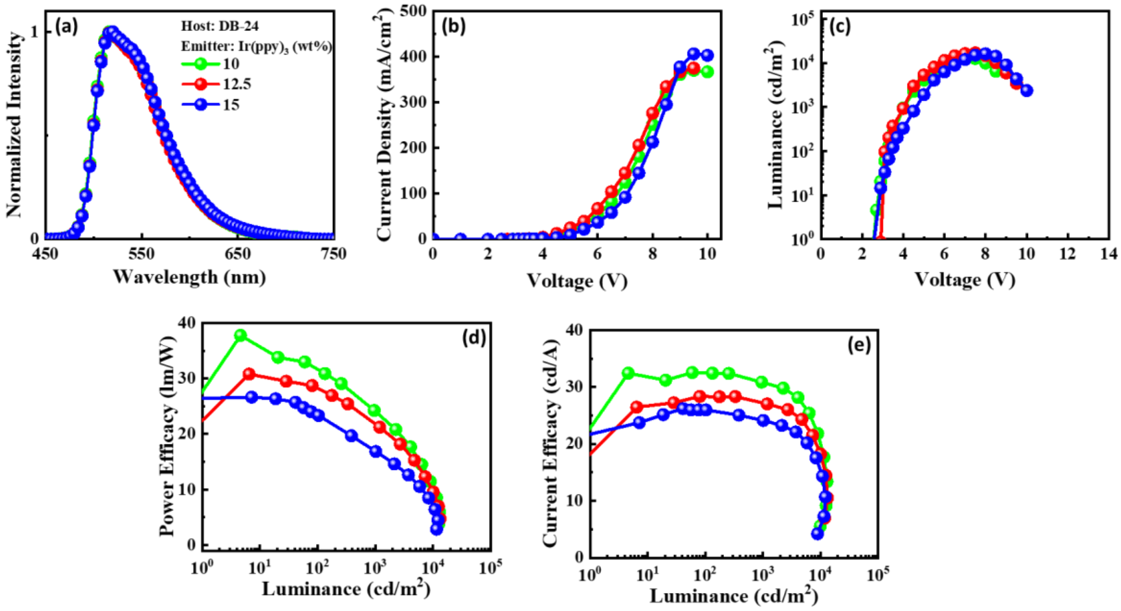

Figure S3. The electroluminescent (EL) properties of the emitter $Ir(ppy)_3$ doped in DB 24 host matrix at varying concentrations showing (**a**) EL spectra, (**b**) current density-voltage, (**c**) luminance-voltage, (**d**) power efficacy-luminance, and (**e**) current efficacy-luminance characteristics.

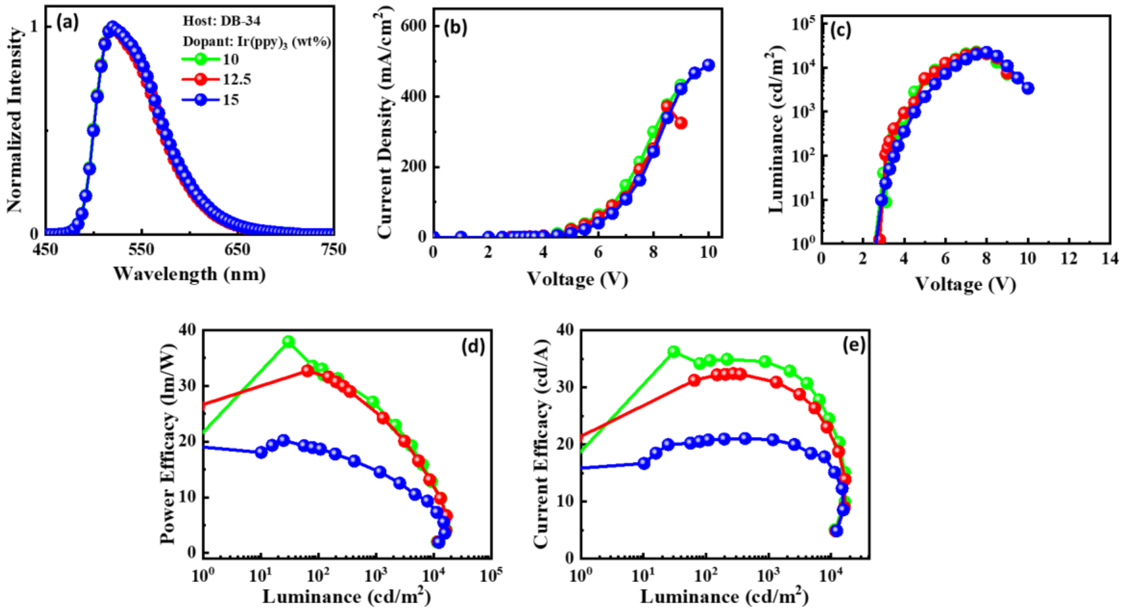

Figure S4. The electroluminescent (EL) properties of the emitter Ir(ppy)3 doped in DB 34 host matrix at varying concentrations showing (**a**) EL spectra, (**b**) current density-voltage, (**c**) luminance-voltage, (**d**) power efficacy-luminance, and (**e**) current efficacy-luminance characteristics.

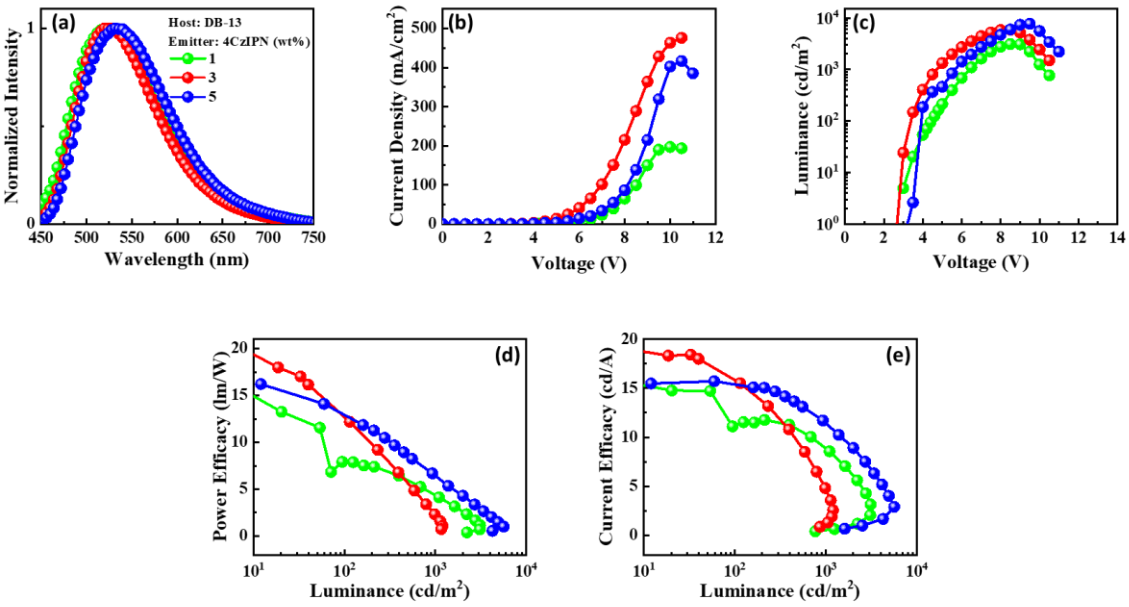

Figure S5. The electroluminescent (EL) properties of the emitter 4CzIPN doped in DB 13 host matrix at varying concentrations showing (**a**) EL spectra, (**b**) current density-voltage, (**c**) luminance-voltage, (**d**) power efficacy-luminance, and (**e**) current efficacy-luminance characteristics.

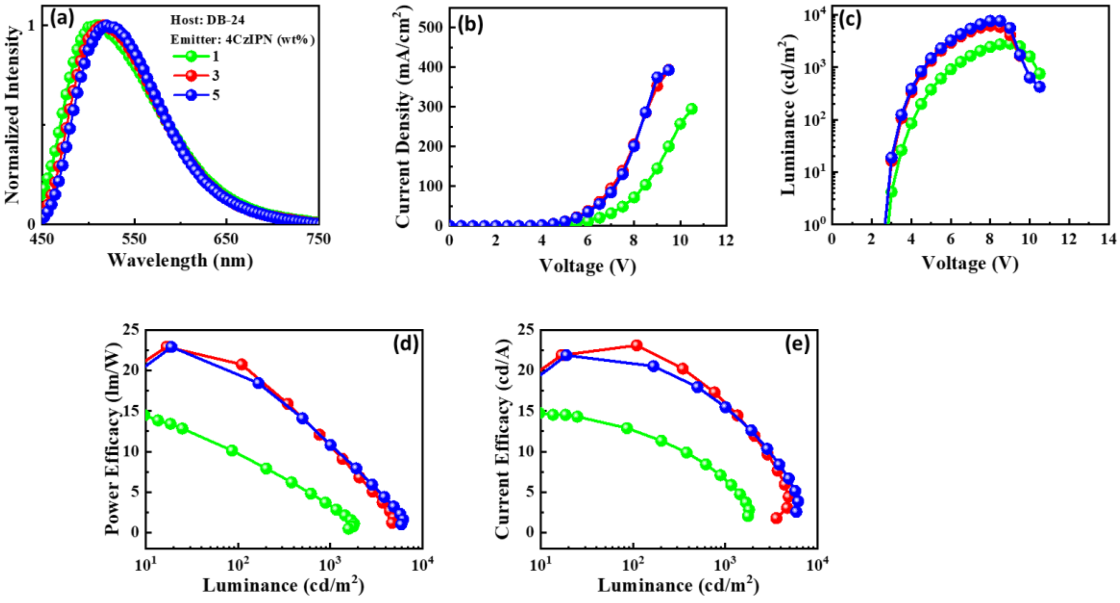

Figure S6. The electroluminescent (EL) properties of the emitter 4CzIPN doped in DB 23 host matrix at varying concentrations showing (**a**) EL spectra, (**b**) current density-voltage, (**c**) luminance-voltage, (**d**) power efficacy-luminance, and (**e**) current efficacy-luminance characteristics.

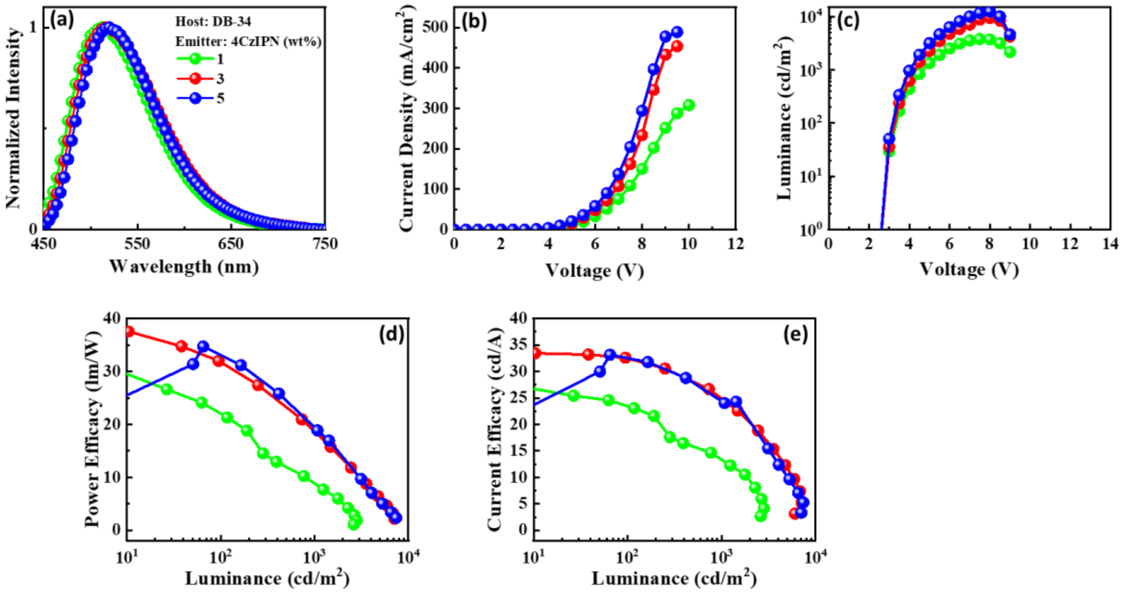

Figure S7. The electroluminescent (EL) properties of the emitter 4CzIPN doped in DB 34 host matrix at varying concentrations showing (**a**) EL spectra, (**b**) current density-voltage, (**c**) luminance-voltage, (**d**) power efficacy-luminance, and (**e**) current efficacy-luminance characteristics.

References

[1] Romero, B.; Schaer, M.; Leclerc, M.; Ades, D.; Siove, A.; Zuppiroli, L. The role of carbazole in organic light-emitting devices. *Synthetic Metals* **1996**, *80*, 271–277. https://doi.org/10.1016/0379-6779(96)80213-X.

[2] Vaitkeviciene, V.; Grigalevicius, S.; Grazulevicius, J.; Jankauskas, V.; Syromyatnikov, V. Hole-transporting [3,3′]bicarbazolyl-based polymers and well-defined model compounds. *European Polymer J.* **2006**, *42*, 2254–2260. https://doi.org/10.1016/j.eurpolymj.2006.06.023.
